# The Medical Reporter

**Published:** 1853-09

**Authors:** 


*The Medical Reporter*, a Quarterly Journal, published under the direction of the Chester and Delaware County Medical Societies. Terms, $1 per annum, in advance.

THE object of this Journal, as indicated in the introduction, is to preserve, in a permanent form, the transactions of the two medical societies, under whose direction it is published. We add it to our exchange list with pleasure. J.

